# Pan-sarcoma genomic analysis of *KMT2A* rearrangements reveals distinct subtypes defined by *YAP1–KMT2A–YAP1* and *VIM*–*KMT2A* fusions

**DOI:** 10.1038/s41379-020-0582-4

**Published:** 2020-05-27

**Authors:** Lucas R. Massoth, Yin P. Hung, Valentina Nardi, G. Petur Nielsen, Robert P. Hasserjian, Abner Louissaint, Adam S. Fisch, Vikram Deshpande, Lawrence R. Zukerberg, Jochen K. Lennerz, Martin Selig, Krzysztof Glomski, Parth J. Patel, Kevin Jon Williams, Ethan S. Sokol, Brian M. Alexander, Jo-Anne Vergilio, Jeffrey S. Ross, Dean C. Pavlick, Ivan Chebib, Erik A. Williams

**Affiliations:** 1 Department of Pathology, Massachusetts General Hospital, Harvard Medical School, Boston, MA 02114 USA; 2 Department of Pathology and Laboratory Medicine, Hartford Hospital, 80 Seymour Street, Hartford, CT 06102 USA; 3 Department of Surgery, Lewis Katz School of Medicine at Temple University, Philadelphia, PA 19140 USA; 4 Department of Physiology and Medicine, Lewis Katz School of Medicine at Temple University, Philadelphia, PA 19140 USA; 5 Foundation Medicine, Inc., 150 Second Street, Cambridge, MA 02141 USA; 6 Department of Pathology, State University of New York Upstate Medical University, 766 Irving Avenue, Syracuse, NY 13210 USA

**Keywords:** Sarcoma, Diagnostic markers

## Abstract

Sarcomas are driven by diverse pathogenic mechanisms, including gene rearrangements in a subset of cases. Rare soft tissue sarcomas containing *KMT2A* fusions have recently been reported, characterized by a predilection for young adults, sclerosing epithelioid fibrosarcoma-like morphology, and an often aggressive course. Nonetheless, clinicopathologic and molecular descriptions of *KMT2A*-rearranged sarcomas remain limited. In this study, we identified by targeted next-generation RNA sequencing an index patient with *KMT2A* fusion-positive soft tissue sarcoma. In addition, we systematically searched for *KMT2A* structural variants in a comprehensive genomic profiling database of 14,680 sarcomas interrogated by targeted next-generation DNA and/or RNA sequencing. We characterized the clinicopathologic and molecular features of *KMT2A* fusion-positive sarcomas, including *KMT2A* breakpoints, rearrangement partners, and concurrent genetic alterations. Collectively, we identified a cohort of 34 sarcomas with *KMT2A* fusions (0.2%), and *YAP1* was the predominant partner (*n* = 16 [47%]). Notably, a complex rearrangement with *YAP1* consistent with *YAP1*–*KMT2A*–*YAP1* fusion was detected in most cases, with preservation of *KMT2A* CxxC-binding domain in the *YAP1*–*KMT2A*–*YAP1* fusion and concurrent deletions of corresponding exons in *KMT2A*. The tumors often affected younger adults (age 20–66 [median 40] years) and histologically showed variably monomorphic epithelioid-to-spindle shaped cells embedded in a dense collagenous stroma. Ultrastructural evidence of fibroblastic differentiation was noted in one tumor examined. Our cohort also included two sarcomas with *VIM*–*KMT2A* fusions, each harboring concurrent mutations in *CTNNB1*, *SMARCB1*, and *ARID1A* and characterized histologically by sheets of spindle-to-round blue cells. The remaining 16 *KMT2A*-rearranged sarcomas in our cohort exhibited diverse histologic subtypes, each with unique novel fusion partners. In summary, *KMT2A*-fusion-positive sarcomas most commonly exhibit sclerosing epithelioid fibrosarcoma-like morphology and complex *YAP1*–*KMT2A–YAP1* fusions. Cases also include rare spindle-to-round cell sarcomas with *VIM–KMT2A* fusions and tumors of diverse histologic subtypes with unique *KMT2A* fusions to non-*YAP1* non-*VIM* partners.

## Introduction

Sarcomas are mesenchymal tumors driven by diverse pathogenic mechanisms, including gene rearrangements in a subset of cases [1]. A fraction of sarcomas remain unclassified or difficult to classify based on histologic and immunohistochemical profile, with no putative driver mutation identified [2]. Recently, oncogenic rearrangements involving *Lysine methyltransferase 2A* (*KMT2A*), a frequent fusion partner in some acute leukemias, have been reported in solid tumors, including types B2 and B3 thymomas and soft tissue sarcomas [3–7]. Yoshida et al. first described two soft tissue sarcomas with *KMT2A* rearrangements, one each to the partners *yes-associated protein 1* (*YAP1)* and *vimentin* (*VIM*) [3]. Two subsequent studies described *YAP1* as a recurrent fusion partner in *KMT2A*-rearranged sarcomas that showed histologic features reminiscent of sclerosing epithelioid fibrosarcoma, and in some cases, low-grade fibromyxoid sarcoma [4, 6]. Nonetheless, clinicopathologic and molecular descriptions of *KMT2A*-rearranged sarcomas remain limited.

In this study, we described an index patient with a sarcoma that showed sclerosing epithelioid fibrosarcoma-like morphology and harbored rearrangements in *KMT2A* and *YAP1*. Prompted by this case and the recent literature, we queried *KMT2A* structural alterations in a comprehensive genomic profiling database of sarcomas and identified 33 additional *KMT2A*-rearranged sarcomas, including 15 additional tumors with similar sclerosing epithelioid fibrosarcoma-like morphology and complex rearrangements with *YAP1*, two cases with spindle-to-round cytomorphology and *VIM–KMT2A* fusions, and 16 sarcomas of diverse histologic subtypes each with unique novel fusion partners with *KMT2A*. We characterized the clinicopathologic and molecular features of *KMT2A* fusion-positive sarcomas, including *KMT2A* breakpoints, rearrangement partners, and concurrent genomic alterations.

## Materials and methods

### Characterization of index patient sarcoma

This part of the study was approved by the Partners Institutional Review Board (Protocol No. 2016P001180). Clinicopathologic and immunophenotypic features were reviewed. Electron microscopy was performed using a FEI Morgagni transmission electron microscope, with images captured with Advanced Microscopy Techniques 2K digital CCD camera, as previously described [8].

Next-generation sequencing-based anchored multiplex PCR was performed for targeted RNA fusion transcript detection as described [9], and targeted sequencing of genomic DNA was performed using Illumina (San Diego, CA) for detection of single nucleotide variants, insertion/deletion (indel), and copy number alterations (lists of gene targets in Supplementary Table 1). Briefly, RNA and DNA were first extracted from formalin-fixed paraffin-embedded tissue (Formapure RNA Isolation, Agencourt AMPure; Beckman Coulter Life Sciences, Indianapolis, IL). In the RNA-based assays, double-stranded cDNA was synthesized, end repaired, adenylated, and ligated with half-functional adapters; two hemi-nested PCR reactions using custom primers from ArcherDx (Boulder, CO) were performed. A laboratory-developed algorithm was used for fusion detection and annotation, with alignment to reference human genome (hg19) using bwa-mem [10].

### Comprehensive genomic analysis and cohort

Comprehensive genomic profiling was performed in a Clinical Laboratory Improvement Amendments-certified, College of American Pathologists-accredited laboratory (Foundation Medicine, Inc., Cambridge, MA, USA). Approval for this study, including a waiver of informed consent and a HIPAA waiver of authorization, was obtained from the Western Institutional Review Board (Protocol No. 20152817). The presence of tumor was confirmed on the hematoxylin and eosin-stained slides prior to nucleic acid extraction and sequencing. DNA and RNA were extracted from 40 μm sections of formalin-fixed paraffin-embedded tissue. Comprehensive genomic profiling was performed using FoundationOne Heme® (F1H), a clinical grade, high-throughput, hybridization capture-based NGS assay for targeted sequencing of all exons of 406 genes, and RNA sequencing of 265 genes. F1H methods used have been previously reported in detail [11–14]. Sequences were analyzed for genomic alterations including short variant alterations, copy number alterations, and gene rearrangements as described [11–14]. Filtering of gene rearrangement candidates was performed by mapping quality (MQ > 30) and distribution of alignment positions (s.d. >10), along with manual review by expert analysts. Tumor mutational burden (mutations/Mb) was determined on 1.2 Mbp of sequenced DNA [13]. For sequence alignment of transcripts, the reference sequences for *KMT2A* and *YAP1* used were NM_005933 and NM_006106, respectively; to be consistent with prior literature, the nomenclature was converted to NM_001197104 and NM_001130145, respectively.

After exclusion of hematologic-related myeloid/monocytic sarcoma, this cohort of non-hematologic soft tissue sarcomas harboring *KMT2A* fusions comprised 33 cases. Clinicopathological data including patient age, gender, tumor site, and stage (based on AJCC 8th edition) [15] were extracted from the accompanying pathology reports, with histologic review of representative hematoxylin and eosin-stained sections of all tumors by IC, YPH, LRM, EAW, and GPN.

## Results

### *KMT2A–YAP1* fusion in the index patient with aggressive soft tissue sarcoma showing sclerosing epithelioid fibrosarcoma-like histology

**Fig. 1:**
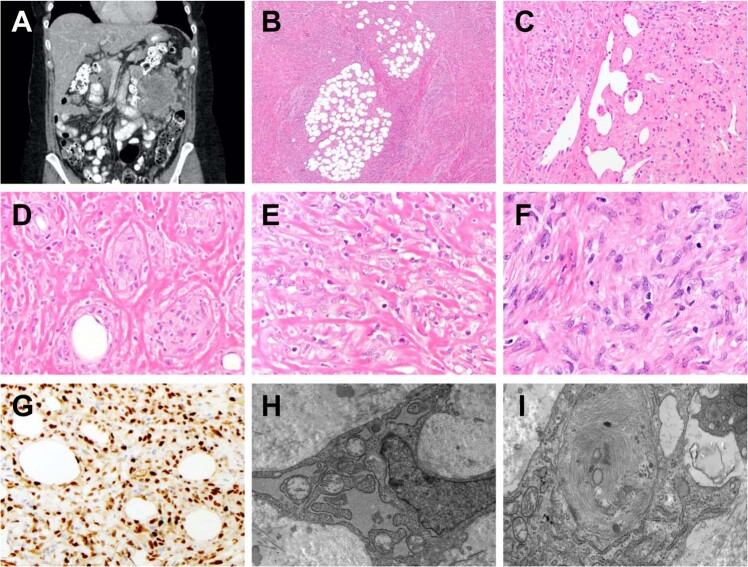
Index patient with *YAP1–KMT2A* fusion-positive sarcoma. **a** Abdominopelvic computed tomography shows a 13.0 cm mass in the left upper quadrant. **b** Histology shows tumor cells infiltrating around fibroadipose tissue, with fibrotic stroma (H&E, 40×). **c** A variably epithelioid-to-spindly region of tumor cells in a sclerotic stroma with gaping vessels and infiltration of eosinophils (H&E, 200×). **d** Tumor cells infiltrate among individual adipocytes and form round aggregates, replacing residual adipocytes surrounded by sclerosis (H&E, 400×). **e** Epithelioid-to-spindle tumor cells contain moderate amounts of eosinophilic cytoplasm, large nuclei, vesicular chromatin, and often prominent nucleoli (H&E, 400×). **f** A focal region of tumor cells shows haphazard arrangement and feathery collagenous stroma (H&E, 400×). **g** By immunohistochemistry, tumor cells show diffuse nuclear cyclin D1 staining (400×). **h**, **i** Ultrastructural analysis shows an extracellular collagenous matrix, abundant dilated endoplasmic reticulum (**h**, 8900×), and small aggregates of filaments (**i**, 14000×).

A 42-year-old woman with no prior medical history presented with a palpable abdominal mass and associated discomfort. Abdominopelvic computed tomography demonstrated a 13.0 cm mass in the left upper quadrant with multiple peritoneal metastases (Fig. 1a). After the diagnostic biopsy (see below), the patient received palliative radiation and died of disease 7 months after presentation.

The biopsy demonstrated an infiltrative sarcoma with spindled-to-epithelioid cytomorphology within a dense hyalinized-to-sclerotic background, histologically reminiscent of sclerosing epithelioid fibrosarcoma. Variable cytologic and architectural patterns were seen, including varying tumor cell density and deceptively bland-appearing pauci-cellular areas (Fig. 1b–f). By immunohistochemistry, tumor cells demonstrated strong nuclear cyclin D1 expression (Fig. 1g) and multifocal smooth muscle actin staining but were negative for MUC4, ERG, CD34, S-100 protein, desmin, STAT6, CD45, MDM2, CDK4, ALK, and ROS1. By electron microscopy, tumor cells showed evidence of fibroblastic differentiation, with prominent dilated rough endoplasmic reticulum, aggregates of filaments, and cohesion as joined by small junctions (Fig. 1h, i). Cytogenetic analysis performed on a fresh tumor sample showed a normal karyotype in 15 metaphases.

Using an RNA-based fusion assay (Supplementary Table 1d), we identified an in-frame oncogenic fusion between *KMT2A* exon 6 (5′) and *YAP1* exon 9 (3′). Due to the primer design, this assay could not assess breakpoints that contain *KMT2A* as a 3′ partner. Additional RNA-based fusion assays (using non-*KMT2A* gene targets; Supplementary Table 1a, b) and targeted DNA-based next-generation sequencing (Supplementary Table 1c) identified no additional pathologic alterations, including *EWSR1* or *FUS* rearrangements.

### Soft tissue sarcomas with complex rearrangements between *YAP1* and *KMT2A*

To assess the overall frequency of *KMT2A* structural alterations in sarcomas, we queried a genomic profiling database of 14,680 sarcomas, in which there was coverage of *KMT2A* by DNA and/or RNA-based sequencing methods. We identified 33 (0.2%) soft tissue sarcomas beyond the index case that harbored *KMT2A* rearrangements. This cohort included 15 additional soft tissue sarcomas with complex rearrangements between *YAP1* and *KMT2A* (Table 1), two soft tissue sarcomas with *VIM–KMT2A* (Supplementary Table 2, patients #S1–S2), and 16 soft tissue sarcomas with *KMT2A* fusions to unique non-*YAP1* non-*VIM* partners (Supplementary Table 2, patients #S3–S18).

**Table 1:** Clinicopathologic and molecular features of 29 *YAP1*–*KMT2A* fusion-positive sarcomas, including 16 from this study (patient cases #1–16) and 13 from prior reports (patient cases #17–29).

Patient no.	Age/gender	Primary location (sequenced site if different)	Position of fusion (5′–3′) breakpoint 1	Position of fusion (5′–3′) breakpoint 2	Concurrent *KMT2A* exon-level deletion	Concurrent genomic alterations	Molecular assay/sequencing method (prior reference, if applicable)
1	42/F	Abdomen	Not evaluated	*KMT2A* exon 6 to *YAP1* exon 9	Not evaluated	None	MGH fusion, MGH snapshot
2	35/F	Unknown (lung)	*YAP1* exon 5 to *KMT2A* exon 4	*KMT2A* exon 6 to *YAP1* exon 9	Not detected	*CDKN2A* homozygous loss, *STAG2* p.R953*, *PTEN* p.D92N, *PASK* c.3814+1G>A	FoundationOne Heme^®^
3	60/F	Abdomen	*YAP1* exon 5 to *KMT2A* exon 4	*KMT2A* exon 6 to *YAP1* exon 9	Not detected	None	FoundationOne Heme^®^
4	31/F	Retroperitoneum	*YAP1* exon 5 to *KMT2A* exon 4	*KMT2A* exon 6 to *YAP1* exon 9	Not detected	*RB1* homozygous loss	FoundationOne Heme^®^
5	66/F	Scalp	*YAP1* exon 5 to *KMT2A* exon 4	*KMT2A* exon 6 to *YAP1* exon 9	Exons 4–6 (DNA and RNA)	Homozygous loss of *CDKN2A* and *CD58*	FoundationOne Heme^®^
6	47/M	Chest	*YAP1* exon 5 to *KMT2A* exon 4	*KMT2A* exon 6 to *YAP1* exon 9	Exons 4–28 (RNA only)	*CDKN2A* homozygous loss	FoundationOne Heme^®^
7	63/F	Abdomen	*YAP1* exon 5 to *KMT2A* exon 4	*KMT2A* exon 6 to *YAP1* exon 9	Exons 4–6 (RNA only)	Amplification of *MDM2* and *PMI1*, *CDKN2A* homozygous loss	FoundationOne Heme^®^
8	33/M	Back (right axilla)	*YAP1* exon 5 to *KMT2A* exon 4	*KMT2A* exon 6 to *YAP1* exon 9	Exons 4–6 (DNA and RNA)	None	FoundationOne Heme^®^
9	29/M	Right leg (thoracic spine)	*YAP1* exon 5 to *KMT2A* exon 4	*KMT2A* exon 6 to *YAP1* exon 9	Exons 4–26 (DNA and RNA)	*CDKN2A* homozygous loss, *FLCN* p.W306*	FoundationOne Heme^®^
10	32/F	Left thigh	*YAP1* exon 5 to *KMT2A* exon 4	*KMT2A* exon 6 to *YAP1* exon 9	Not detected	Amplification of *MDM4*, *IKBKE*, and *MCL1*	FoundationOne Heme^®^
11	20/F	Unknown (pleura)	*YAP1* exon 5 to *KMT2A* exon 4	*KMT2A* exon 6 to *YAP1* exon 9	Exons 4–6 (DNA and RNA)	*CDKN2A* homozygous loss, *ZNF703* amplification	FoundationOne Heme^®^
12	64/F	Left hand (lung)	*YAP1* exon 5 to *KMT2A* exon 4	*KMT2A* exon 6 to *YAP1* exon 9	Exons 4–6 (RNA only)	*CD36* p.V263fs*16	FoundationOne Heme^®^
13	38/M	Left hip (pleura)	*YAP1* exon 5 to *KMT2A* exon 4	*KMT2A* exon 6 to *YAP1* exon 9	Exons 4–6 (DNA and RNA)	*CDKN2A* homozygous loss	FoundationOne Heme^®^
14	30/F	Peritoneum (right shoulder)	*YAP1* exon 5 to *KMT2A* exon 4	*KMT2A* exon 6 to *YAP1* exon 9	Not detected	Homozygous loss of *CDKN2A* and *RB1*, *ATR* duplication intron 15—exon 20	FoundationOne Heme^®^
15	54/M	Right buttock (lung)	*YAP1* exon 3 to *KMT2A* exon 5	*KMT2A* exon 6 to *YAP1* exon 5	Exons 5–6 (DNA and RNA)	None	FoundationOne Heme^®^
16	52/M	Right buttock (lung)	*YAP1* exon 3 to *KMT2A* exon 5	*KMT2A* exon 6 to *YAP1* exon 5	Not detected	Amplification of *MDM2* and *FRS2*	FoundationOne Heme^®^
17	20/F	Thigh	*YAP1* exon 5 to *KMT2A* exon 4	*KMT2A* to *YAP1* (exons not reported)	N/A	N/A	RNA-sequencing (Yoshida et al. case 1)
18	45/F	Paraspinal	*YAP1* exon 5 to *KMT2A* exon 4	*KMT2A* exon 6 to *YAP1* exon 9	N/A	N/A	RNA-sequencing (Kao et al. case 1)
19	45/F	Thigh	*YAP1* exon 4 to *KMT2A* exon 5	*KMT2A* exon 5 to *YAP1* exon 9^a^	N/A	N/A	RNA-sequencing (Kao et al. case 2)
20	47/M	Lower leg	*YAP1* exon 5 to *KMT2A* exon 4	*KMT2A* exon 6 to *YAP1* exon 9	N/A	N/A	RNA-sequencing (Kao et al. case 3)
21	42/M	Thigh	*YAP1* exon 5 to *KMT2A* exon 4	Not detected	N/A	N/A	RT-PCR (Kao et al. case 8)
22	22/M	Neck/paraspinal	Not detected	*KMT2A* exon 6 to *YAP1* exon 9	N/A	N/A	Targeted DNA NGS (Kao et al. case 9)
23	91/F	Finger	*YAP1* exon 6 to *KMT2A* exon 5^b^	*KMT2A* exon 6 to *YAP1* exon 9	N/A	N/A	RNA-sequencing (Puls et al. case 1)
24	11/M	Supraclavicular fossa	*YAP1* exon 5 to *KMT2A* exon 4	*KMT2A* exon 6 to *YAP1* exon 9	N/A	N/A	RNA-sequencing (Puls et al. case 2)
25	16/F	Heel/ankle	*YAP1* exon 3 to *KMT2A* exon 5	*KMT2A* exon 6 to *YAP1* exon 8	N/A	N/A	RNA-sequencing (Puls et al. case 3)
26	53/M	Foot	*YAP1* exon 5 to *KMT2A* exon 4	*KMT2A* exon 6 to *YAP1* exon 9	N/A	N/A	RNA-sequencing (Puls et al. case 4)
27	69/M	Chest wall	Not detected	*KMT2A* exon 6 to *YAP1* exon 9	N/A	N/A	RNA-sequencing (Puls et al. case 5)
28	51/F	Thigh	*YAP1* exon 4 to *KMT2A* exon 5	*KMT2A* exon 6 to *YAP1* exon 9	N/A	N/A	RNA-sequencing (Puls et al. case 6)
29	40/M	Thigh	*YAP1* exon 4 to *KMT2A* exon 5	*KMT2A* exon 6 to *YAP1* exon 9	N/A	N/A	RNA-sequencing (Puls et al. case 7)

Data are presented using transcripts *YAP1* NM_001130145 and *KMT2A* NM_001197104 (see “Methods”). Breakpoints in cases #1–16 are reported based on the highest read count.

^a^Transcript out-of-frame; breakpoints reported to be within exons.

^b^Breakpoint inside *KMT2A* exon 5 (AA 1143) involving the so-called “pre-CxxC” domain (1115–1154) that accounts for a portion of the multivalent bond with PAFc.

Collectively, among the 16 patients with *YAP1*–*KMT2A* fusion-positive sarcomas, 56% were female, and ages ranged from 20 to 66 (median 40) years. Primary tumor locations included abdomen/peritoneum/retroperitoneum (36%), hip/back/buttock (29%), upper or lower extremities (21%), and chest/scalp (14%). This cohort included 13 patients with stage IV (81%), one patient with stage III (6%), and two patients with stage II disease (13%). Of sixteen tumors, the submitting pathologic diagnoses included “sclerosing epithelioid fibrosarcoma” in five, “sclerosing fibrosarcoma” in one, and descriptive diagnoses that included “sarcoma,” “favor sarcoma,” or “spindle cell neoplasm” in the remainder ten cases (including the descriptor “epithelioid” in six cases and “fibrosarcoma” in one case).

**Fig. 2:**
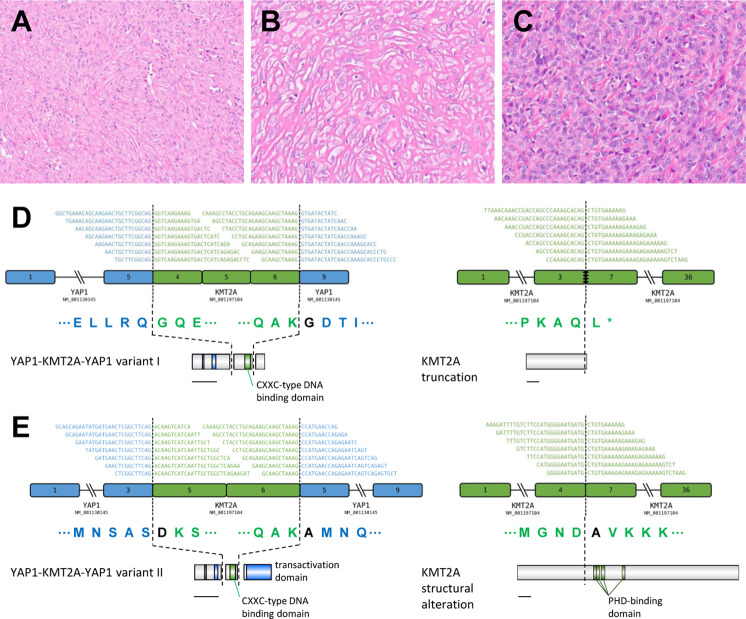
Histologic and Molecular Features of *YAP1–KMT2A* fusion-positive sarcomas. **a**–**c** Representative histology of three *YAP1*–*KMT2A* fusion-positive sarcomas with variably monomorphic epithelioid-to-spindle cells, morphologically reminiscent of sclerosing epithelioid fibrosarcoma (**a** H&E, 200×; **b**, **c** H&E, 400×). **d**, **e** Schematic of complex *YAP1*–*KMT2A–YAP1* rearrangement, with variant I (**d**) and variant II (**e**) fusions along with co-occurring *KMT2A* deletions.

Histology of this cohort (available for 15 of 16 tumors) demonstrated features resembling sclerosing epithelioid fibrosarcoma in all tumors, with variably monomorphic epithelioid-to-spindle cells embedded in a collagenous stroma (Fig. 2a–c). Characteristic features of low-grade fibromyxoid sarcoma, such as arcade-like vessels and variably prominent myxoid background, were absent. The index case was remarkable for a small region with feathery-appearing stromal collagen and showed staghorn hemangiopericytoma-like vasculature multifocally. Accompanying pathology reports specified that immunohistochemistry for MUC4 was negative in all four tumors tested, and that break-apart FISH for *EWSR1* rearrangements was negative in all four tested cases.

Fusions between *YAP1* and *KMT2A* were detected by RNA-based assays in all 16 sarcomas, six of which were additionally confirmed by orthogonal DNA-based assays (Table 1). In each of the 15 sarcomas in which both the 5′ and 3′ breakpoints of *KMT2A* could be evaluated, two sets of breakpoints involving *KMT2A* and *YAP1* were identified. In 13 tumors (patients #2–14), the first sets of breakpoints corresponded to fusion of *YAP1* exon 5 to *KMT2A* exon 4, and the second sets of breakpoints corresponded to fusion of *KMT2A* exon 6 to *YAP1* exon 9 (variant I), indicative of insertion of *KMT2A* exons 4–6 into *YAP1* and loss of *YAP1* exons 6–8. Of these, eight tumors had separate *KMT2A* deletions of exons 4–6 detected at the DNA and/or RNA level (Table 1).

In the remaining two tumors (patients #15–16), the first sets of breakpoints corresponded to fusion of *YAP1* exon 3 to *KMT2A* exon 5, and the second sets of breakpoints corresponded to fusion of *KMT2A* exon 6 to *YAP1* exon 5 (variant II), indicative of insertion of *KMT2A* exons 5–6 into *YAP1* between exons 3 and 5. In one of these two tumors, separate *KMT2A* deletion of exon 5–6 was detected.

No gene amplification of *KMT2A* was identified. Concurrent genomic alterations included homozygous loss of *CDKN2A* in eight tumors, homozygous loss of *RB1* in two tumors, and *MDM2* amplification in two tumors (Table 1). Median tumor mutational burden (TMB) was 1.2 mutations/Mb (range <0.8–5.7).

### Soft tissue sarcomas with *VIM*–*KMT2A* fusion

**Fig. 3:**
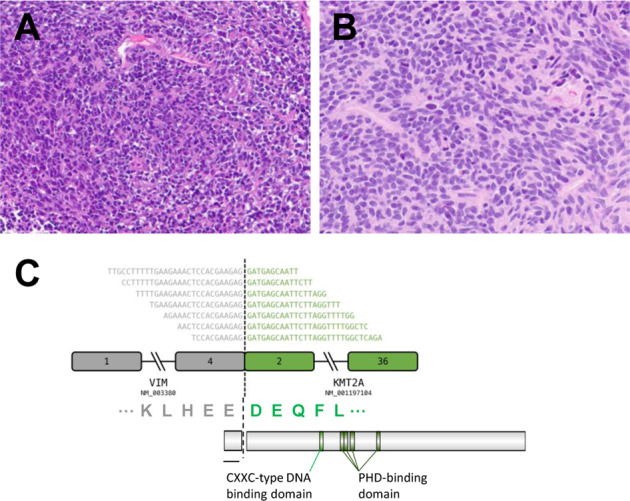
Histologic and molecular features of *VIM–KMT2A* fusion-positive sarcomas. **a**, **b** Representative histology of two *VIM–KMT2A* sarcomas with spindle-to-round cell morphology (**a** H&E, 200×; **b** H&E, 400×). **c** Schematic of *VIM–KMT2A* rearrangement.

Two soft tissue sarcomas with *VIM*–*KMT2A* fusion were identified (Supplementary Table 2, patients #S1–S2); both occurred in men, aged 43 and 46 years, and involved the lower extremities. Histologically, both were round and spindle cell sarcomas, characterized by monomorphic spindled-to-round cells with moderate amounts of eosinophilic cytoplasm (Fig. 3a, b). *VIM* was identified as the 5′ fusion partner to *KMT2A*, with breakpoints near exon 4 and exon 2 respectively (Fig. 3c). Both tumors harbored concurrent mutations in *CTNNB1*, *SMARCB1*, and *ARID1A* (Supplementary Table 2). TMB was 0.8 and 1.6 mutations/Mb.

### Diverse soft tissue sarcomas with *KMT2A* fusion to non-*YAP1* Non-*VIM* partners

**Fig. 4:**
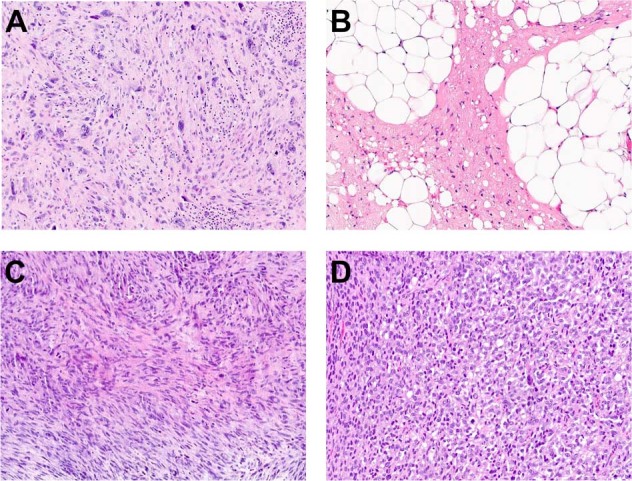
Histologic features of sarcomas with nonrecurrent *KMT2A* rearrangements. **a** Giant-cell rich pleomorphic sarcoma (case #S5; H&E, 200×). **b** Atypical lipomatous tumor (case #S10; H&E, 200×). **c** Undifferentiated pleomorphic sarcoma (case #S13; H&E, 200×). **d** High-grade endometrial stromal sarcoma (case #S17; H&E, 200×).

Sixteen tumors of diverse histologic subtypes were identified with *KMT2A* fusion to nonrecurrent partners (Supplementary Table 2, patients #S3–S18). These tumors affected patients across a wide age range (6–70 [median 57] years) and encompassed diverse histologic subtypes, including leiomyosarcoma, atypical lipomatous tumor, and undifferentiated pleomorphic sarcoma (Fig. 4a–d), among others. None of these 16 tumors demonstrated histologic features of sclerosing epithelioid fibrosarcoma or low-grade fibromyxoid sarcoma. *KMT2A* was the 3′ fusion partner in eight tumors and the 5′ fusion partner in seven tumors, with no reciprocal transcripts identified in any of the cases. The remaining tumor (patient #S7) showed separate fusion partners to *KMT2A* 5′ and 3′ fragments. Pathogenic *TP53* mutation was the most common co-occurring mutation, present in seven (44%) tumors in this group. Median TMB was 3.2 mutations/Mb (range <0.8–8.1).

## Discussion

In this study, we described 34 soft tissue sarcomas with *KMT2A* rearrangements. *YAP1* is a commonly recurrent partner to *KMT2A*, and *YAP1*–*KMT2A* fusion-positive sarcomas exhibit sclerosing epithelioid fibrosarcoma-like histomorphology. We found that *YAP1*–*KMT2A* rearrangements are complex in configuration, showing two reciprocally oriented breakpoints and often concurrent *KMT2A* exon deletions, altogether in keeping with a complex *YAP1*–*KMT2A*–*YAP1* fusion. We also described the features of *VIM*–*KMT2A* sarcomas as well as sarcomas of diverse histologic subtypes that harbored unique nonrecurrent *KMT2A* fusions of unclear significance.

### Sarcomas containing complex rearrangements of *YAP1* and *KMT2A*

This study augments the clinicopathologic descriptions of *YAP1*–*KMT2A* fusion-positive sarcomas [3, 4, 6]. *YAP1*–*KMT2A* fusion-positive sarcomas may behave aggressively, given the rapid decline in the index patient and the advanced tumor stage in most cases. A limitation of our study is that the queried database is enriched for aggressive cases and advanced disease where genetic testing is desired for therapeutic target identification. From 11 patients with available follow-up in the three prior studies, three showed no recurrence after initial resection, five recurred locally or with unresectable tumors, and three harbored metastatic disease; altogether, three of 11 reported patients died of disease [3, 4, 6].

*YAP1*–*KMT2A* fusion-positive sarcomas contained histologic patterns reminiscent of sclerosing epithelioid fibrosarcoma. While low-grade fibromyxoid sarcoma-like histology was noted in three of seven cases in one study [6], this was not observed in other studies [3, 4]. Our histologic review, limited to a representative section in most cases, precluded full assessment of such “hybrid” features. However, none of the tumors received a histologic diagnosis of low-grade fibromyxoid sarcoma rendered by the submitting pathologist. Immunohistochemical expression of cyclin D1, as noted in the index case, might represent a diagnostic clue. The lack of MUC4 expression in the four tested tumors herein was also noted previously in all 13 sequencing-confirmed cases [3, 4, 6]. We additionally provided the first description of the ultrastructural features of *YAP1*–*KMT2A* fusion-positive sarcoma, which appeared similar to those reported in sclerosing epithelioid fibrosarcoma [16, 17]. Sclerosing epithelioid fibrosarcoma is characterized by MUC4 expression and recurrent *EWSR1–CREB3L1* or *FUS–CREB3L2* fusion in most cases [18]. While MUC4 status may be a useful discriminator between *EWSR1*/*FUS*-rearranged sclerosing epithelioid fibrosarcoma and *YAP1–KMT2A* fusion-positive tumors, additional studies to determine the performance of this immunohistochemical marker would be needed for definitive assessment. Furthermore, identification of *YAP1*–*KMT2A* fusion-positive soft tissue sarcomas with sclerosing epithelioid sarcoma-like histologic and ultrastructural features raises the question of whether these sarcomas should be classified as a distinct entity related to or within the spectrum of sclerosing epithelioid fibrosarcoma.

Detection of the complex *YAP1*–*KMT2A* rearrangement can be challenging. In prior studies, concurrent *KMT2A* and *YAP1* rearrangements were noted in only one tumor tested by both break-apart fluorescence in situ hybridization (FISH) probes [3]; negative FISH results for *KMT2A* and/or *YAP1* were common in cases where the fusion was confirmed by sequencing [4]. FISH testing is thus not sensitive for identifying *YAP1*–*KMT2A* fusion-positive sarcoma. Other detection methods include targeted next-generation DNA-based or RNA-based sequencing assays. While *KMT2A* is frequently included on the sequencing panels for hematolymphoid neoplasms, it is less often covered on solid tumor sequencing panels due to the low prevalence of *KMT2A* structural alterations in solid tumors, including sarcomas. Nonetheless, our findings suggest that cases with sclerosing epithelioid fibrosarcoma-like histology that lack *EWSR1* and *FUS* rearrangements may be selected for additional testing that includes *KMT2A* or *YAP1* coverage.

While prior studies have generally assumed the expression of two separate reciprocal transcripts and hypothesized the *YAP1–KMT2A* fusion alone to be pathogenic [3, 4, 6], published data show frequently discrepant sets of breakpoints between the *YAP1–KMT2A* transcripts and the *KMT2A–YAP1* transcripts (see below), suggesting a complex rearrangement. Based on our analysis, we hypothesize a novel *YAP1*–*KMT2A*–*YAP1* configuration (Fig. 2d, e), which may be accounted for by a “cut-and-paste” structural mechanism in some cases [19].

This complex *YAP1–KMT2A–YAP1* fusion configuration is supported by multiple lines of reasoning and evidence. First, *YAP1* is located 16 Mb centromeric to *KMT2A* on the same plus strand on chromosome 11q; reciprocal translocations between *YAP1* and *KMT2A* could not be accounted for by a single intrachromosomal inversion event. Deletion between 5′ *YAP* and 3′ *KMT2A* could also generate an in-frame fusion but would not account for the two sets of fusion breakpoints observed. We detected two sets of reciprocally oriented breakpoints involving *KMT2A* and *YAP1* in every fully evaluated case, along with frequent concurrent exon deletion of *KMT2A* involving the same exons implicated in the complex fusion. In fact, a review of all previously published *YAP1*–*KMT2A* fusion-positive sarcomas with reported breakpoints confirmed our observations, with *KMT2A* exons 5–6 (or exons 4–6) present in all predicted full-length transcripts and shared between sets of reciprocals (Table 1; patients #17–29), except in patient case #19; this lone exception demonstrated an out-of-frame 5′ *KMT2A* transcript lacking exon 6, similar to a low-level splice variant seen in the index case absent of *KMT2A* exon 6. Otherwise, the inclusion of *KMT2A* exons 5–6 (which encodes the CxxC-binding domain) within a *YAP1*–*KMT2A*–*YAP1* fusion characterized all eight prior cases reporting both breakpoints, similar to current cases [3, 4, 6].

Second, given that the *KMT2A* CxxC-binding domain is integral to *HOX* gene regulation, it is notable that no increase in *HOXA*-related gene expression was detected by RNA sequencing in three *YAP1–KMT2A* fusion-positive sarcomas in a prior study [4]. *KMT2A* (formerly *MLL*) belongs to the polymerase associated factor complex (PAFc) [20], which modifies chromatins and regulates target genes such as *HOX* [21–23]. In ~10% of acute leukemias in which *KMT2A* is fused to one of >80 fusion partners [20, 24], *KMT2A* fusion breakpoints are located primarily within exons 8–14 (the so-called “breakpoint cluster region”) [20, 22, 25], preserving both the CxxC-binding domain in exons 5–6 and the adjacent repression domain 2 (RD2) largely encoded by exons 7–8. The latter is required for interaction with PAFc and engagement of transcription machinery at downstream targets critical to leukemogenesis, including *HOXA* cluster genes [22, 25]. The lack of *HOXA*-related gene expression in those three *YAP1–KMT2A* fusion-positive sarcomas argues against a simple *YAP1–KMT2A* fusion that preserves these domains, but rather a complex *YAP1–KMT2A–YAP1* fusion that retains the CxxC-binding domain but lacks the RD2 domain. Given the importance of the CxxC-binding domain in the pathogenesis of acute leukemias, the consistent retention of the CxxC-binding domain in *YAP1–KMT2A–YAP1* fusion suggests that it plays a key role in the pathogenesis of these sarcomas [25, 26].

In addition to *KMT2A* exons 5–6, the *YAP1–KMT2A–YAP1* construct retains at least *YAP1* exons 1–3 and exon 9, with the former encoding the TEAD-binding domain. This domain is responsible for the downstream activation of TEAD transcription factors documented in other *YAP1*-related oncogenic fusions [27]. *YAP1* exon 9 contains a PDZ-binding motif that has been demonstrated to play a role in some forms of *YAP1*-mediated oncogenesis, which is required for TEAD-associated transcription of the cell proliferation gene *connective tissue growth factor* (*CCN2/CTGF*) [28]. Interestingly, *CCN2* encodes for a matricellular protein that is ubiquitously overexpressed in fibrotic diseases, and overexpression in fibroblasts is sufficient to cause fibrosis in tissues of multiple types [29].

### Sarcomas with *VIM–KMT2A* rearrangements and other nonrecurrent partners to *KMT2A*

Among the remaining *KMT2A* rearrangements in this study, only the *VIM–KMT2A* fusion was recurrent (*n* = 2) and was associated with small round-to-spindle histology. The only previously published *VIM–KMT2A* fusion-positive sarcoma [3] showed similar histology and expressed NKX2.2 and EMA, both of which were also expressed in one of our cases herein and may represent potential diagnostic adjuncts. Collectively, these three *VIM–KMT2A* sarcomas presented in young adult males in the lower extremities. *VIM*, located on chromosome 10p13, encodes vimentin and has been described in fusions with *FOS* in a subset of epithelioid hemangioma [30, 31]. The initial study on a *VIM–KMT2A* sarcoma demonstrated high expression of *KMT2A* and the downstream target *HOXA* genes [3], likely driven by the highly active *VIM* promoter in the *VIM*–*KMT2A* fusion, with retention of both CxxC-binding and RD2 domains in *KMT2A* that allow PAFc-associated chromatin modification of *HOX*-related genes.

In the remaining sarcomas with *KMT2A* fusions to non-*YAP1* non-*VIM* partners, none of the gene partners were recurrent or previously described, and these sarcomas harbored diverse histologic diagnoses. While leiomyosarcoma was the most common diagnosis, there was no clear unifying theme, and none showed sclerosing epithelioid fibrosarcoma-like histology as in *YAP1–KMT2A* fusion-positive sarcomas or small round blue cell-like histology in *VIM*–*KMT2A* fusion-positive sarcomas. The rarity of nonrecurrent *KMT2A* fusions in histologically diverse sets of sarcomas underscores the importance of correlating molecular results with clinicopathologic parameters when reaching the diagnosis. Given their rarity and the frequent presence of diverse co-occurring mutations, these nonrecurrent *KMT2A* fusions may represent sequelae of genomic instability rather than bona fide oncogenic drivers. The fusions have not been corroborated by orthogonal techniques, and the significance of these nonrecurrent fusions should be interpreted with caution.

In conclusion, in a comprehensive genomic profiling database of 14,680 sarcomas, *KMT2A* rearrangements were observed in 0.2% of cases. Most *KMT2A* fusions involved complex rearrangements with *YAP1*, consistent with a *YAP1*–*KMT2A*–*YAP1* fusion configuration. *YAP1–KMT2A* fusion-positive sarcomas primarily affected young adults and showed a sclerosing epithelioid fibrosarcoma-like histology. *VIM*–*KMT2A* sarcomas also showed a predilection for young adults, however with a spindle-to-round cell morphology. Other unique and nonrecurrent *KMT2A* fusions occurred in sarcomas of diverse histologic subtypes with unclear significance. Identification of sarcomas with pathogenic *KMT2A* fusions raises a possibility of targeted therapies which are actively being pursued in *KMT2A*-rearranged leukemias [32, 33]. Comprehensive genomic profiling of sarcomas may aid the characterization of recurrent complex alterations and enable precise tumor classification in conjunction with their clinicopathologic contexts.

## Supplementary information

Supplemental Table 1

Supplemental Table 2

## References

[1] Fletcher C, Bridge J, Hogendoorn P, Mertens F (2013). WHO classification of tumours of soft tissue and bone. Pathology and genetics of tumours of soft tissue and bone.

[2] Mastrangelo G, Coindre JM, Ducimetière F, Dei Tos AP, Fadda E, Blay JY (2012). Incidence of soft tissue sarcoma and beyond: a population-based prospective study in 3 European regions. Cancer..

[3] Yoshida A, Arai Y, Tanzawa Y, Wakai S, Hama N, Kawai A (2019). KMT2A (MLL) fusions in aggressive sarcomas in young adults. Histopathology..

[4] Kao YC, Lee JC, Zhang L, Sung YS, Swanson D, Hsieh TH (2019). Recurrent YAP1 and KMT2A gene rearrangements in a subset of MUC4-negative sclerosing epithelioid fibrosarcoma. Am J Surg Pathol.

[5] Watson S, Perrin V, Guillemot D, Reynaud S, Coindre JM, Karanian M (2018). Transcriptomic definition of molecular subgroups of small round cell sarcomas. J Pathol..

[6] Puls F, Agaimy A, Flucke U, Ploegmakers M, Stoehr R, Kindblom L (2020). Recurrent fusions between YAP1 and KMT2A in morphologically distinct neoplasms within the spectrum of low-grade fibromyxoid sarcoma and sclerosing epithelioid fibrosarcoma. Am J Surg Pathol.

[7] Massoth LR, Hung YP, Dias-santagata D, Onozato M, Shah N (2020). Pan-Cancer landscape analysis reveals recurrent KMT2A-MAML2 gene fusion in aggressive histologic subtypes of thymoma. JCO Precis Oncol..

[8] Massoth LR, Selig MK, Little BP, Chebib I, Kradin RL (2019). Multiple calcifying fibrous pseudotumors of the pleura: ultrastructural analysis provides insight on mechanism of dissemination. Ultrastruct Pathol.

[9] Zheng Z, Liebers M, Zhelyazkova B, Cao Y, Panditi D, Lynch KD (2014). Anchored multiplex PCR for targeted next-generation sequencing. Nat Med..

[10] Li H, Durbin R (2009). Fast and accurate short read alignment with Burrows-Wheeler transform. Bioinformatics..

[11] Frampton GM, Fichtenholtz A, Otto GA, Wang K, Downing SR, He J (2013). Development and validation of a clinical cancer genomic profiling test based on massively parallel DNA sequencing. Nat Biotechnol..

[12] Sun JX, He Y, Sanford E, Montesion M, Frampton GM, Vignot S (2018). A computational approach to distinguish somatic vs. germline origin of genomic alterations from deep sequencing of cancer specimens without a matched normal. PLoS Pathog..

[13] Chalmers ZR, Connelly CF, Fabrizio D, Gay L, Ali SM, Ennis R (2017). Analysis of 100,000 human cancer genomes reveals the landscape of tumor mutational burden. Genome Med.

[14] He J, Abdel-Wahab O, Nahas MK, Wang K, Rampal RK, Intlekofer AM (2016). Integrated genomic DNA/RNA profiling of hematologic malignancies in the clinical setting. Blood..

[15] Amin MB, Edge S, Greene F, Byrd DR, Brookland RK, Washington MK, et al. AJCC cancer staging system. 8th ed. New York: Springer; 2017.

[16] Jiao YF, Nakamura SI, Sugai T, Uesugi N, Habano W, Ogata M (2002). Overexpression of MDM2 in a sclerosing epithelioid fibrosarcoma: genetic, immunohistochemical and ultrastructural study of a case. Pathol Int.

[17] Donner LR, Clawson K, Dobin SM (2000). Sclerosing epithelioid fibrosarcoma: a cytogenetic, immunohistochemical, and ultrastructural study of an unusual histological variant. Cancer Genet Cytogenet.

[18] Arbajian E, Puls F, Magnusson L, Thway K, Fisher C, Sumathi VP (2014). Recurrent EWSR1–CREB3L1 gene fusions in sclerosing epithelioid fibrosarcoma. Am J Surg Pathol.

[19] Li Y, Roberts ND, Wala JA, Shapira O, Schumacher SE, Kumar K (2020). Patterns of somatic structural variation in human cancer genomes. Nature..

[20] Winters AC, Bernt KM (2017). MLL-rearranged leukemias—an update on science and clinical approaches. Front Pediatr..

[21] Milne TA, Briggs SD, Brock HW, Martin ME, Gibbs D, Allis CD (2002). MLL targets SET domain methyltransferase activity to Hox gene promoters. Mol Cell..

[22] Tan J, Muntean AG, Hess JL (2010). PAFc, a key player in MLL-rearranged leukemogenesis abstract. Oncotarget.

[23] Butler LH, Slany R, Cui X, Cleary ML, Mason DY (1997). The HRX proto-oncogene product is widely expressed in human tissues and localizes to nuclear structures. Blood..

[24] Meyer C, Burmeister T, Gröger D, Tsaur G, Fechina L, Renneville A (2018). The MLL recombinome of acute leukemias in 2017. Leukemia..

[25] Muntean AG, Tan J, Sitwala K, Huang Y, Bronstein J, Connelly JA (2010). The PAF complex synergizes with MLL fusion proteins at HOX loci to promote leukemogenesis. Cancer Cell..

[26] Ayton PM, Chen EH, Cleary ML (2004). Binding to nonmethylated CpG DNA is essential for target recognition, transactivation, and myeloid transformation by an MLL oncoprotein. Mol Cell Biol.

[27] Sekine S, Kiyono T, Ryo E, Ogawa R, Wakai S, Ichikawa H (2019). Recurrent YAP1-MAML2 and YAP1-NUTM1 fusions in poroma and porocarcinoma. J Clin Investig.

[28] Shimomura T, Miyamura N, Hata S, Miura R, Hirayama J, Nishina H (2014). The PDZ-binding motif of Yes-associated protein is required for its co-activation of TEAD-mediated CTGF transcription and oncogenic cell transforming activity. Biochem Biophys Res Commun.

[29] Jun J, Lau L (2011). Taking aim at the extracellular matrix. Nat Rev Drug Discov.

[30] van Ijzendoorn DGP, de Jong D, Romagosa C, Picci P, Benassi MS, Gambarotti M (2015). Fusion events lead to truncation of FOS in epithelioid hemangioma of bone. Genes Chromosom Cancer.

[31] Huang SC, Zhang L, Sung YS, Chen CL, Krausz T, Dickson BC (2015). Frequent FOS gene rearrangements in epithelioid hemangioma: a molecular study of 58 cases with morphologic reappraisal. Am J Surg Pathol.

[32] Somers K, Wen VW, Middlemiss SMC, Osborne B, Forgham H, Jung MS (2019). A novel small molecule that kills a subset of MLL-rearranged leukemia cells by inducing mitochondrial dysfunction. Oncogene..

[33] Chan AKN, Chen CW (2019). Rewiring the epigenetic networks in MLL-rearranged leukemias: epigenetic dysregulation and pharmacological interventions. Front Cell Dev Biol.

